# The clinical prognosis of patients with cN0 papillary thyroid microcarcinoma by central neck dissection

**DOI:** 10.1186/s12957-015-0553-2

**Published:** 2015-04-07

**Authors:** Liyang Zhang, Ziwen Liu, Yuewu Liu, Weisheng Gao, Chaoji Zheng

**Affiliations:** General Surgery Department, Peking Union Medical College Hospital, Chinese Academy of Medical Sciences, No.1 Shuaifu Garden, Dongcheng District, 100730 Beijing, China

**Keywords:** Papillary thyroid microcarcinoma (PTMC), Central lymph node dissection (CLND), Prognosis

## Abstract

**Background:**

Central lymph node metastasis of papillary thyroid microcarcinoma (PTMC) is common; however, prophylactic central lymph node dissection (CLND) is still controversial because of the possible increased morbidity. The purpose of this study is to determine the clinical outcome of patients with cN0 PTMC by central neck dissection.

**Methods:**

A retrospective cohort study was conducted on patients with PTMC without preoperative evidence of lymph node disease (cN0), and the outcomes were compared between patients undergoing total thyroidectomy (TT) alone (group A) and patients undergoing TT with CLND (group B).

**Results:**

In this study, 242 patients with cN0 PTMC were included. Group A had 108 patients and group B had 134 patients. During a follow-up of over 60 months, the long-term postoperative complications were equivalent between the two groups. In group B, the presence of involved central neck lymph nodes upstaged 16% of patients to stage III disease, which necessitated additional postoperative radioactive iodine treatment. More patients had recurrences in group A. The rate of reoperation in the central compartment was higher in group A than in group B (8.3% *vs* 2.2%, *P* < 0.01).

**Conclusions:**

Prophylactic CLND does not increase long-term postoperative complications and reduces the risk of recurrence in the central compartment.

## Background

Despite the majority of papillary thyroid microcarcinoma (PTMC) patients having benign clinical courses, metastases to the lymph nodes are common. A report from Mayo Clinic showed that one third of 900 PTMC patients had metastases at the time of diagnosis [1]. The central neck compartment is the most common site of metastases. Consequently, some surgeons recommend routine prophylactic removal of the central neck lymph nodes at the time of the initial operation [2,3]. However, this was countered with some studies showing no benefit [4,5]. The purpose of this study is to evaluate the influence of prophylactic central lymph node dissection (CLND) on the oncological and functional outcomes of patients with PTMC.

## Methods

This retrospective cohort study was performed of a single institutional database of patients with histologically proven PTMC (<1 cm) at Peking Union Medical College Hospital from January 2008 to December 2009. Patients who underwent total thyroidectomy (TT) alone were designated as group A, whereas those who underwent TT with prophylactic CLND were designated as group B. Some surgeons in our institution performed routine CLND, and these surgeons contributed patients to group B. Those who did not perform CLND routinely contributed patients to group A. Patients with bilateral PTMC underwent bilateral prophylactic CLND, while patients with unilateral PTMC underwent ipsilateral CLND. CLND is defined as the removal of all level VI and VII nodes [6]. Patients with common papillary thyroid carcinoma (PTC) of >1 cm, evident preoperative abnormal lymph nodes, distant metastasis, or previous operation for PTC were excluded. Patients who underwent other operations such as lobectomy or subtotal thyroidectomy were also excluded in order to keep homogeneity in the management of patients and to avoid interference on cancer recurrence. Of the 709 patients treated surgically for PTC from January 2008 to December 2009, 242 patients satisfied the inclusion criteria. Of these, 108 patients underwent TT alone and 134 patients underwent TT and CLND. Consent statements were required from all patients.

We routinely removed badly blood-supported parathyroid during operation and cut it into 1-mm pieces to implant in the sternocleidomastoid muscle. Patients were monitored postoperatively with serum calcium determination. Temporary hypocalcemia was defined as serum calcium <8 mg/dl anytime during the initial 6-month follow-up. Permanent hypoparathyroidism was defined as a need for continued calcium beyond 6 months after surgery with persistent serum calcium <8 mg/dl. Transient recurrent laryngeal nerve (RLN) palsy was confirmed by fiber optic laryngoscopy between 0 and 6 months after operation, and permanent RLN palsy was confirmed by fiber optic laryngoscopy beyond 6 months after operation. Recurrence was defined as local or regional disease requiring reoperation or other repeat treatment, as detected by serial cervical ultrasonographies or radioactive thyroid scan [6].

Patients underwent radioactive iodine ablative therapy with iodine^131^ (I^131^) 3 to 4 weeks after operation. The indications for I^131^ ablation were determined by the nuclear medicine specialists based on the American Joint Committee on Cancer (AJCC) stage of disease [7]. All patients entered a scheduled clinical follow-up program on an average duration of over 60 months. Follow-up of patients involved annual examination of neck ultrasonography, I^131^ scintigraphy, computed tomography, and so on.

Statistical analysis was performed using SPSS13.0 software. Data were compared for statistical analysis using the chi-square test to evaluate differences between qualitative variables and using Student’s *t* test to compare quantitative variables. *P* < 0.05 was considered significant.

## Results

Patient characteristics are summarized and operative results were analyzed between group A and group B based on operative and surgical pathology reports in Table 1. There were no significant differences in the age, gender distribution, and tumor pathologic characteristics. In group B, ipsilateral CLND was performed in 112 (84%) patients and 31 (16%) had bilateral CLND. The number of patients found to have involved central neck lymph nodes on final pathology was 51 of 134 (38%) in group B. The median number of lymph nodes excised was 0 in group A and 6 in group B (*P* < 0.01). The median number of positive lymph nodes was 0 in group A and 1 in group B (*P* < 0.01). A total of 22 (16%) patients over 45 years old were upstaged from stage I to stage III disease because of positive lymph node status according to the AJCC staging system for differential thyroid cancer.

**Table 1:** **Patient characteristics**

	**Group A (n = 108)**	**Group B (n = 134)**	***P*** **value**
Median age	45	48	NS
Gender			
Male	27	26	NS
Female	81	108	NS
Pathology			
Median tumor size (cm)	0.5	0.7	NS
Extrathyroid extension	2	4	NS
Median nodes removed	0	6	<0.01
Median positive nodes	0	1	<0.01
Median MACIS score	3.8	4.1	NS
Median follow-up (months)	66	61	NS

NS, not significant.

The incidence of operative complications is summarized in Table 2. The incidence of postoperative infection and hematoma was similar between the groups. There were a significantly greater number of parathyroid auto-transplantations due to devascularization and a higher rate of temporary postoperative hypocalcemia in group B. Permanent hypoparathyroidism was equivalent between the groups. There was no difference in the incidence of temporary and permanent RLN injury between group A and group B. We did not list the complications of CLND in the reintervention because the number was too small to make a statistical analysis and compare it with that of the initial operation. There were 12 recurrences in both groups, and 8 of them had re-CLND, 2 of whom had nerve injury and 3 developed permanent hypocalcemia, which was much higher than that of the initial operation.

**Table 2:** **Operative complications**

	**Group A (n = 108)**	**Group B (n = 134)**	***P*** **value**
Wound infection	0	0	NS
Hemorrhage	0	1	NS
Temporary hypocalcemia	10 (9%)	40 (30%)	<0.01
Permanent hypoparathyroidism	0	2	NS
Temporary RLN injury	1 (0.9%)	2 (1.5%)	NS
Permanent RLN injury	1 (0.9%)	1 (0.7%)	NS
Mean parathyroids autotransplanted	0.1	0.65	<0.01

NS, not significant.

**Figure 1:**
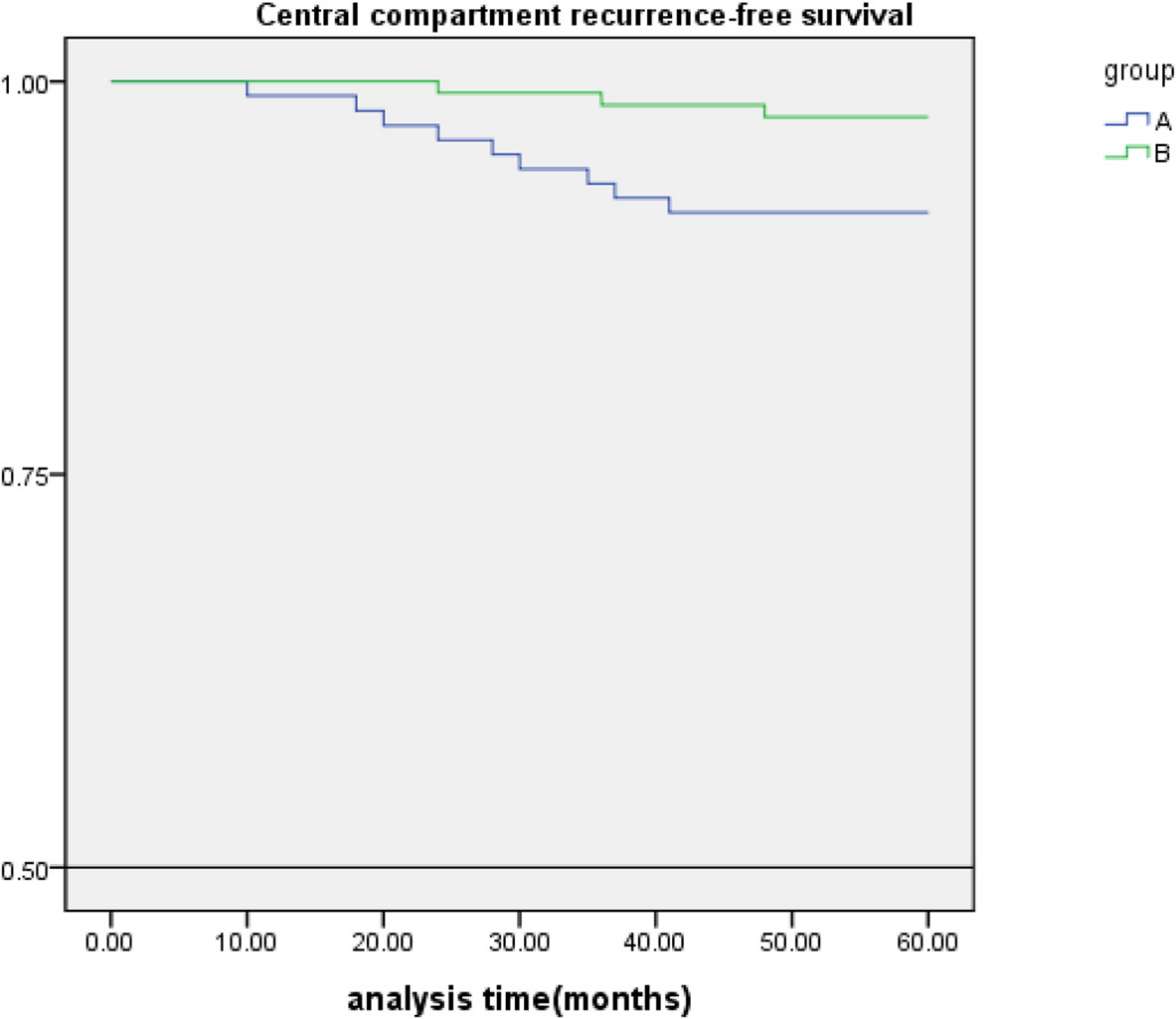
**Central compartment recurrence-free survival.**

During the final follow-up, no one died in this study. A Kaplan-Meier survival analysis for central compartment reoperation was undertaken. Group B, which had prophylactic CLND, had a significantly lower failure rate (*P* < 0.05) compared with group A, which did not have CLND (shown in Figure 1). There was no recurrence in the thyroid bed. All 12 recurrences occurred in regional lymph nodes, 4 of whom had both central and lateral section lymph node recurrences. Recurrent disease occurred in 9 of 108 (8.3%) in group A and 3 of 134 (2.2%) in group B (*P* < 0.01). In 8 central compartment lymph node (levels VI and VII) recurrences, 7 (6.5%) was in group A and 1 (0.7%) was in group B (*P* < 0.01). In 8 lateral section lymph node (levels IV, III, and II) recurrences, 5 (4.6%) was in group A and 3 (2.2%) was in group B (NS). Eight of 12 recurrences had re-CLND (levels VI and VII), 4 of whom had both central (levels VI and VII) and lateral (levels IV, III, and II) section reoperation. The median time to reoperation in group B was longer than that in group A (27 *vs* 18 months, *P* < 0.05). The rate of reoperation in the central compartment was significantly higher in group A than in group B (6.5% *vs* 0.7%, *P* < 0.01). Although the rate of reoperation in the lateral section in group B (2.2%) was lower than that in group A (4.6%), there was no statistical significance. Data summarizing reoperation rates and regions of lymph nodes removed at secondary surgery are shown in Table 3.

**Table 3:** **Disease recurrence requiring reoperation**

	**Group A (%)**	**Group B (%)**	***P*** **value**
Median time to reoperation (months)	18	27	<0.05
Reoperation	9 (8.3%)	3 (2.2%)	<0.01
Central	7 (6.5%)	1 (0.7%)	<0.01
Lateral	5 (4.6%)	3 (2.2%)	NS
Central plus lateral	3 (2.8%)	1 (0.7%)	<0.01

NS, not significant.

## Discussion

The incidence of PTMC has been increasing within the recent 5 years with the extensive use of thyroid ultrasonography in routine physical examination [8]. Although it has been accepted that patients with PTMC have a good prognosis [9], cervical lymph node metastases are common, with an incidence between 30% and 60% [1,10]. Furthermore, more and more researches reported that regional lymph node metastasis was associated with increased local recurrence rates and reduced survival [11,12]. Therefore, there is renewed interest in the operative management of cervical lymph node metastases, but controversies remain. Most surgeons agree that grossly involved lymph nodes in the central neck of patients with PTMC should be managed by selective neck dissection. The debate focuses on whether the use of prophylactic CLND at the initial thyroid operation benefits PTMC patients. This retrospective cohort study examined the impact of routine CLND in addition to TT in the management of cN0 PTMC and suggests a decreased recurrence rate and a reduction in the need for reoperation in the central compartment without increasing long-term postoperative complications.

Prophylactic CLND is defined as the removal of all level VI and VII nodes when there is no evidence of lymph node metastasis on preoperative clinical examination, imaging studies, or intra-operative visual inspection [6]. It has been reported that most of central lymph node metastases are too small to be detected by sonography [13,14]. We observed that approximately 40% of the patients who underwent central neck dissection had lymph node involvement despite negative preoperative physical examination and ultrasonography in our study. The presence of lymph node metastases upgraded 16% of the patients above 45 years old with PTMC from stage I to stage III for central neck dissection, which provided improved staging accuracy rather than for the removal of subclinical disease. It is important to note that accurate staging is not only helpful for predicting the prognosis but also crucial for determining which patients would need radioiodine treatment because for PTMC patients without lymph node metastases, radioiodine therapy is not necessary.

Another advantage of prophylactic central lymph node dissection is that it provides a clear surgical field for central compartment lymph node dissection in the initial operation. In contrast, reoperation after the development of recurrence is a much more difficult and technically challenging procedure, associated with an increased morbidity.

Higher rates of complications such as temporary hypocalcemia, permanent hypoparathyroidism, and RLN palsy are often cited in arguments against prophylactic CLND in the treatment of PTMC [15,16]. Temporary hypoparathyroidism has been reported to be between 20% and 50% [17-19]. Our comparison of the two groups confirms previous reports of increased temporary hypocalcemia with CLND, which was caused by transient parathyroid dysfunction due to devascularization and could be corrected by calcium supplementation. The rate of permanent hypoparathyroidism had no difference between the two groups because of our routine use of intra-operative parathyroid auto-transplantation. The rates of temporary and permanent RLN injury did not increase with prophylactic CLND.

Despite high rates of temporary hypocalcemia, the patients in our study did get benefits from CLND. The removal of subclinical metastases improved the recurrence rate and avoided reoperation in the central compartment, which may be associated with increased risks. As expected, the rate of disease recurrence in group B is lower than that in group A (2.2% *vs* 8.3%, *P* < 0.01). Most of these recurrences were in the first 3 years after the initial operation. The median time of reoperation in group B was also longer than that in group A (27 *vs* 18 months, *P* < 0.05). There was also a reduction in the need for further surgery in the central compartment in patients who had prophylactic CLND in comparison with those who did not have (6.5% *vs* 0.7%, *P* < 0.01). Although the rate of reoperation in the lateral neck in group B is also lower than that in group A, there is no statistically significant difference. Shen et al. have shown a similar trend toward decreased recurrence in patients undergoing prophylactic CLND [20].

The limitations of this study include its nonrandomized, retrospective design, leaving the results vulnerable to selection biases. The strengths are its large population and long-time follow-up.

## Conclusions

In conclusion, this study evaluates the impact of prophylactic CLND in addition to TT in the management of patients with PTMC and shows that prophylactic CLND for cN0 PTMC can be performed at the time of the initial operation without apparent increase in long-term morbidity. Prophylactic CLND should be considered because it not only allows for more accurate staging by the determination of lymph node status but also results in lower recurrence rates of PTMC and a reduction in the need for reoperation.
